# High-throughput screening of Mucoromycota fungi for production of low- and high-value lipids

**DOI:** 10.1186/s13068-018-1070-7

**Published:** 2018-03-14

**Authors:** Gergely Kosa, Boris Zimmermann, Achim Kohler, Dag Ekeberg, Nils Kristian Afseth, Jerome Mounier, Volha Shapaval

**Affiliations:** 1 Faculty of Science and Technology, Norwegian University of Life Sciences, P.O. Box 5003, 1432 Ås, Norway; 2 Faculty of Chemistry, Biotechnology and Food Science, Norwegian University of Life Sciences, P.O. Box 5003, 1432 Ås, Norway; 3 Nofima AS, Osloveien 1, 1433 Ås, Norway; 4 Université de Brest, EA3882 Laboratoire Universitaire de Biodiversité et Ecologie Microbienne, IBSAM, ESIAB, Technopôle Brest Iroise, 29280 Plouzané, France

**Keywords:** High-throughput screening, Mucoromycota, Filamentous fungi, Single cell oil, PUFA, Biodiesel, FTIR

## Abstract

**Background:**

Mucoromycota fungi are important producers of low- and high-value lipids. *Mortierella alpina* is used for arachidonic acid production at industrial scale. In addition, oleaginous Mucoromycota fungi are promising candidates for biodiesel production. A critical step in the development of such biotechnological applications is the selection of suitable strains for lipid production. The aim of the present study was to use the Duetz-microtiter plate system combined with Fourier transform infrared (FTIR) spectroscopy for high-throughput screening of the potential of 100 Mucoromycota strains to produce low- and high-value lipids.

**Results:**

With this reproducible, high-throughput method, we found several promising strains for high-value omega-6 polyunsaturated fatty acid (PUFA) and biodiesel production purposes. Gamma-linolenic acid content was the highest in *Mucor fragilis* UBOCC-A-109196 (24.5% of total fatty acids), and *Cunninghamella echinulata* VKM F-470 (24.0%). For the first time, we observed concomitant gamma-linolenic acid and alpha-linolenic acid (up to 13.0%) production in psychrophilic *Mucor flavus* strains. Arachidonic acid was present the highest amount in *M. alpina* ATCC 32222 (41.1% of total fatty acids). Low cultivation temperature (15 °C) activated the temperature sensitive ∆17 desaturase enzyme in *Mortierella* spp., resulting in eicosapentaenoic acid production with up to 11.0% of total fatty acids in *M. humilis* VKM F-1494. *Cunninghamella blakesleeana* CCM-705, *Umbelopsis vinacea* CCM F-539 and UBOCC-A-101347 showed very good growth (23–26 g/L) and lipid production (7.0–8.3 g/L) with high palmitic and oleic acid, and low PUFA content, which makes them attractive candidates for biodiesel production. *Absidia glauca* CCM 451 had the highest total lipid content (47.2% of biomass) of all tested strains. We also demonstrated the potential of FTIR spectroscopy for high-throughput screening of total lipid content of oleaginous fungi.

**Conclusions:**

The use of Duetz-microtiter plate system combined with FTIR spectroscopy and multivariate analysis, is a feasible approach for high-throughput screening of lipid production in Mucoromycota fungi. Several promising strains have been identified by this method for the production of high-value PUFA and biodiesel.

**Electronic supplementary material:**

The online version of this article (10.1186/s13068-018-1070-7) contains supplementary material, which is available to authorized users.

## Background

Oleaginous microorganisms have been considered for nearly a century as an alternative source for the production of low- and high-value lipids (i.e. single cell oils). However, it is only in the past two or three decades they have been used commercially [1]. Oil of microalgae and filamentous fungi are good sources of high value omega-3 and omega-6 long-chain polyunsaturated fatty acids, respectively. These PUFAs include eicosapentaenoic acid, (EPA, C20:5n3), docosahexaenoic acid (DHA, C22:6n3), γ-linolenic acid (GLA, C18:3n6), dihomo-γ-linolenic acid (DGLA, C20:3n6) and arachidonic acid (ARA, C20:4n6). More than 60% of GLA and ARA of total fatty acids in fungal oil has been reported [2, 3]. ARA produced by *Mortierella alpina* is included in infant formulas worldwide. This fatty acid is necessary for the proper brain and eye development of babies and ARA also prevents the undesirable retro-conversion of DHA to EPA in these formulas [4]. DGLA was reported to possess antitumor properties [5], while GLA has been used to alleviate premenstrual tension and for the improvement of various skin conditions [4, 6]. Recently, microbial lipids (yeasts, filamentous fungi and microalgae) have been considered as possible alternative source for biodiesel production, since they can potentially contain high amounts of saturated (SAT) and monounsaturated fatty acids (MUFA) and can grow rapidly in a controlled environment. The commercially produced single cell oil contains high amount of PUFA, and the process is based on heterotrophic cultivation, where the most often used substrate is glucose [1, 7]. However, for low-value biodiesel application, low cost substrates, such as food rest materials, waste glycerol and lignocellulosic materials are being tested for their economical sustainability. Interestingly, fungi (yeast and molds) are able to grow and accumulate lipids on such substrates [8–11].

Many members of Mucoromycota fungi have been reported as oleaginous [8, 12, 13]. Ratledge performed extensive screening of more than 300 Mucoromycota fungi (13 genera) based on several criteria to find the best GLA producer [7]. A *Mucor circinelloides* strain was selected and the industrial production of GLA started with this strain in 1985 [7]. Similarly, Weete et al. screened more than 150 Mucoromycota strains for GLA production and showed that *Syzygites megalocarpus* accumulated up to 62% GLA in the oil [3]. Eroshin et al. [14] and Botha et al. [15] performed screening of 87 and 61 *Mortierella* strains, respectively, for ARA production in agar medium, and *M. alpina* was shown as the best producer. All the studies cited above were specifically focused on the production of high-value fatty acids and in most cases, on a single high-value PUFA. In addition, screening in the latter studies were performed in a shake flask/bioreactor/agar plate set-up, often without statistically relevant number of replicates [3, 14, 16–19]. To our best knowledge, the extensive evaluation of Mucoromycota fungi (with three biological replicates) for the production of a broad spectrum of low- and high-value lipids for different applications has not been performed so far.

Miniaturization of fermentation technologies has enabled the screening a high number of strains under controlled conditions [20, 21]. Recently, we demonstrated the reproducible high-throughput cultivation of oleaginous filamentous fungi in Duetz-microtiter plate system (Duetz-MTPS) [22, 23]. In addition, we showed that FTIR spectroscopy combined with multivariate analyses, is a powerful high-throughput analytical approach for the quantitative and qualitative assessment of total lipid content, lipid classes and individual fatty acids in the fungal biomass [23, 24]. A precise quantitative measurement of extracellular metabolites and nutrients in the cultivation medium was also obtained [22].

The aim of this study was to perform the screening of 100 strains of Mucoromycota fungi including *Amylomyces*, *Mucor*, *Rhizopus*, *Umbelopsis*, *Absidia*, *Lichtheimia*, *Cunninghamella* and *Mortierella* species, for their ability to produce low and high-value lipids by combining cultivation in Duetz-MTPS with FTIR analysis of fungal biomass.

## Methods

### Fungal strains

One hundred Mucoromycota strains, belonging to three families and eight genera, i.e., *Mucor*, *Amylomyces*, *Rhizopus*, *Umbelopsis*, *Absidia*, *Cunninghamella*, *Lichtheimia* and *Mortierella* were used in this study (Table 1 and Additional file 1: Figure S1). Fungi were obtained in agar slants and dishes or in lyophilized form, from the Czech Collection of Microorganisms (CCM; Brno, Czech Republic), the Food Fungal Culture Collection (FRR; Commonwealth Scientific and Industrial Research Organisation, North Ryde, Australia), the Norwegian School of Veterinary Science (VI; Oslo, Norway), the Université de Bretagne Occidentale Culture Collection (UBOCC; Plouzané, France), the All-Russian Collection of Microorganisms (VKM; Moscow, Russia) and the American Type Culture Collection (ATCC; VA, USA).

**Table 1:** List of Mucoromycota strains used for the screening of lipid production

No.	Strains	No.	Strains
1	*Mucor circinelloides* VI 04473	51	*Rhizopus stolonifer* VKM F-399
2	*Mucor circinelloides* CCM 8328	52	*Rhizopus stolonifer* VKM F-400
3	*Mucor circinelloides* FRR 4846	53	*Umbelopsis isabellina* UBOCC-A-101350
4	*Mucor circinelloides* FRR 5020	54	*Umbelopsis isabellina* UBOCC-A-101351
5	*Mucor circinelloides* FRR 5021	55	*Umbelopsis isabellina* VKM F-525
6	*Mucor circinelloides* UBOCC-A-102010	56	*Umbelopsis ramanniana* CCM F-622
7	*Mucor circinelloides* UBOCC-A-105017	57	*Umbelopsis ramanniana* VKM F-502
8 (II)	*Mucor flavus* CCM 8086	58	*Umbelopsis vinacea* CCM 8333
9 (I)	*Mucor flavus* VKM F-1003	59 (I)	*Umbelopsis vinacea* CCM F-513
10 (I)	*Mucor flavus* VKM F-1097	60	*Umbelopsis vinacea* CCM F-539
11	*Mucor flavus* VKM F-1110	61	*Umbelopsis vinacea* UBOCC-A-101347
12	*Mucor fragilis* CCM F-236	62	*Absidia coerulea* CCM 8230
13	*Mucor fragilis* UBOCC-A-109196	63	*Absidia coerulea* VKM F-627
14	*Mucor fragilis* UBOCC-A-113030	64	*Absidia coerulea* VKM F-833
15	*Mucor hiemalis* FRR 5101	65	*Absidia cylindrospora* CCM F-52T
16	*Mucor hiemalis* UBOCC-A-101359	66	*Absidia cylindrospora* VKM F-1632
17	*Mucor hiemalis* UBOCC-A-101360	67	*Absidia cylindrospora* VKM F-2428
18	*Mucor hiemalis* UBOCC-A-109197	68	*Absidia glauca* CCM 450
19	*Mucor hiemalis* UBOCC-A-111119	69	*Absidia glauca* CCM 451
20	*Mucor hiemalis* UBOCC-A-112185	70	*Absidia glauca* CCM F-444
21	*Mucor lanceolatus* UBOCC-A-101355	71	*Absidia glauca* UBOCC-A-101330
22	*Mucor lanceolatus* UBOCC-A-109193	72	*Lichtheimia corymbifera* CCM 8077
23	*Mucor lanceolatus* UBOCC-A-110148	73	*Lichtheimia corymbifera* VKM F-507
24	*Mucor mucedo* UBOCC-A-101353	74	*Lichtheimia corymbifera* VKM F-513
25	*Mucor mucedo* UBOCC-A-101361	75	*Cunninghamella blakesleeana* CCM F-705
26	*Mucor mucedo* UBOCC-A-101362	76	*Cunninghamella blakesleeana* VKM F-993
27	*Mucor plumbeus* CCM F-443	77	*Cunninghamella echinulata* VKM F-439
28	*Mucor plumbeus* FRR 2412	78	*Cunninghamella echinulata* VKM F-470
29	*Mucor plumbeus* FRR 4804	79	*Cunninghamella echinulata* VKM F-531
30	*Mucor plumbeus* UBOCC-A-109204	80	*Mortierella alpina* ATCC 32222
31	*Mucor plumbeus* UBOCC-A-109208	81	*Mortierella alpina* UBOCC-A-112046
32	*Mucor plumbeus* UBOCC-A-109210	82	*Mortierella alpina* UBOCC-A-112047
33	*Mucor plumbeus* UBOCC-A-111125	83 (IV)	*Mortierella elongata* VKM F-1614
34	*Mucor plumbeus* UBOCC-A-111128	84	*Mortierella elongata* VKM F-524
35	*Mucor plumbeus* UBOCC-A-111132	85 (III)	*Mortierella gamsii* VKM F-1402
36	*Mucor racemosus* CCM 8190	86 (V)	*Mortierella gamsii* VKM F-1529
37	*Mucor racemosus* FRR 3336	87 (III)	*Mortierella gamsii* VKM F-1641
38	*Mucor racemosus* FRR 3337	88 (IV)	*Mortierella gemmifera* VKM F-1252
39	*Mucor racemosus* UBOCC-A-102007	89 (III)	*Mortierella gemmifera* VKM F-1631
40	*Mucor racemosus* UBOCC-A-109211	90	*Mortierella gemmifera* VKM F-1651
41 (II)	*Mucor racemosus* UBOCC-A-111127	91 (V)	*Mortierella globulifera* VKM F-1408
42	*Mucor racemosus* UBOCC-A-111130	92 (V)	*Mortierella globulifera* VKM F-1448
43	*Amylomyces rouxii* CCM F-220	93	*Mortierella globulifera* VKM F-1495
44	*Rhizopus microsporus* CCM F-718	94 (III)	*Mortierella humilis* VKM F-1494
45	*Rhizopus microsporus* CCM F-792	95	*Mortierella humilis* VKM F-1528
46	*Rhizopus microsporus* VKM F-1091	96 (III)	*Mortierella humilis* VKM F-1611
47	*Rhizopus oryzae* CCM 8075	97	*Mortierella hyalina* UBOCC-A-101349
48	*Rhizopus oryzae* CCM 8076	98	*Mortierella hyalina* VKM F-1629
49	*Rhizopus oryzae* CCM 8116	99	*Mortierella hyalina* VKM F-1854
50	*Rhizopus stolonifer* CCM F-445	100	*Mortierella zonata* UBOCC-A-101348

Unless stated otherwise, standard cultivation conditions were used: 28 °C, 90 g/L glucose, 5 days, Duetz-MTPS. Non-standard cultivation conditions: I: 20 °C, II: 15 °C, III: 15 °C, 7 days, IV: 50 g/L glucose, V: 15 °C, 50 g/L glucose, 9 days, shake flask

### Media and growth conditions

Fungal strains were first cultivated on malt extract (MEA) or potato dextrose agar (PDA) for 7 days at 15–25 °C. The majority of the one hundred tested fungi were mesophilic and grew well at room temperature (20–25 °C) with some exceptions (e.g. *Mucor flavus* CCM 8086), which only grew at 15 °C. Spores were then harvested from the agar cultures using a sterile saline solution.

A liquid medium was prepared according to the protocol described by Kavadia et al. [25] with the following modifications (g L^−1^): glucose 50–90, yeast extract 5, KH_2_PO_4_ 7, Na_2_HPO_4_ 2, MgSO_4_·7H_2_O 1.5, CaCl_2_·2H_2_O 0.1, FeCl_3_·6H_2_O 0.008, ZnSO_4_·7H_2_O 0.001, CoSO_4_·7H_2_O 0.0001, CuSO_4_·5H_2_O 0.0001, MnSO_4_·5H_2_O 0.0001. All chemicals were obtained from Merck (Darmstadt, Germany), except yeast extract (Oxoid, Basingstoke, England). The medium pH was 6.05 after sterilization. Spore suspensions (10–100 μL, depending on sporulation strength) were transferred to 2.5 mL liquid medium in 24-square polypropylene deep well plates using the Duetz-MTPS (Enzyscreen, Heemstede, Netherlands) [23]. Inoculated microtiter plates were mounted on an Innova 40R refrigerated desktop shaker (Eppendorf, Hamburg, Germany) using the clamp system and were cultivated with a shaking rate of 300 rpm (circular orbit 0.75”) for 5–7 days at 15–28 °C. Three strains (*Mortierella gamsii* VKM F-1529, *Mortierella globulifera* VKM F-1408 and *Mortierella globulifera* VKM F-1448) failed to grow in the Duetz-MTPS and were grown for 9 days at 15 °C in 500 mL baffled shake flasks (SFs) filled with 100 mL of the above-described medium.

### Experimental design

For each strain, three biological replicates were prepared. Biological replicates were represented by the spore suspensions prepared from separate agar plates. Exceptions were *M. circinelloides* strains with five biological replicates and *M. gamsii*, *M. globulifera* strains, for which only one culture in SF was prepared. To have enough biomass for gas chromatography (GC) analysis, three wells in the MTP were inoculated for each strain and each biological replicate (i.e. eight strains were tested per MTP). In addition, microcultivation of each biological replicate was performed in a separate MTP. After cultivation, biomass from the three wells of each MTP was merged and used for gas chromatography-flame ionization detector (GC-FID), gas chromatography–mass spectrometry (GC–MS) fatty acid analyses and FTIR spectroscopy. The residual glucose content of the supernatant of the growth medium was analyzed by high-performance liquid chromatography (HPLC).

### Microscopy

Micrographs were obtained from fresh biomass according to Kosa et al. [23] in bright-field and fluorescence mode after Nile-red staining with a DM6000B microscope (Leica Microsystems, Wetzlar, Germany).

### Preparation of fungal biomass for HTS–FTIR analysis

Fermentation broth was vacuum filtered on Whatman No. I filter paper (GE Whatman, Maidstone, UK) and the fungal biomass was washed thoroughly with distilled water. Approximately, 10 mg of the washed biomass was transferred into 2 mL screw-cap tube, 500 μL distilled water and 250 ± 30 mg acid-washed glass beads (800 μm, OPS Diagnostics, NJ, USA) were added, then the biomass was homogenized for 1–2 min in a FastPrep-24 high-speed benchtop homogenizer (MP Biomedicals, USA) at 6.5 m s^−1^. This homogenized fungal suspension was used for FTIR analysis.

### FTIR spectroscopy

FTIR analysis of homogenized fungal biomass was performed with the High Throughput Screening eXTension (HTS-XT) unit coupled to the Vertex 70 FTIR spectrometer (both Bruker Optik, Ettlingen, Germany) in transmission mode [23]. The FTIR system was equipped with a globar mid-IR source and a DTGS detector. The spectra were recorded on 384-well silicon microplates in transmission mode, with a spectral resolution of 4 cm^−1^ and digital spacing of 1.928 cm^−1^. Background (reference) spectra of an empty microplate well was recorded before each sample well measurement. The spectra were collected in the 4000–500 cm^−1^ spectral range, with 64 scans for both background and sample spectra, and using an aperture of 5.0 mm. Measurements were controlled by the OPUS 7.5 software (Bruker Optik, Ettlingen, Germany).

### Lipid extraction from the fungal biomass

Washed fungal biomass was frozen at − 20 °C and then lyophilized overnight in an Alpha 1–2 LDPlus freeze-dryer (Martin Christ, Germany) at − 55 °C and 0.01 mbar pressure. Freeze-dried biomass was used to determine biomass concentration (g cell dry weight/L, CDW). Lipid extraction from freeze-dried fungal biomass was based on a cell disruption step with glass beads followed by a direct transesterification-extraction procedure. The detailed method can be found in [23].

### GC-FID total lipid content and fatty acid analysis

Determination of total lipid content of fungal biomass (expressed as the wt% of total fatty acid methyl esters, FAMEs of cell dry weight) and fatty acid composition (expressed as wt% of individual FAME of total FAMEs) analysis were performed with a HP 6890 gas chromatograph (Hewlett Packard, Palo Alto, USA) equipped with an SGE BPX70, 60.0 m × 250 μm × 0.25 μm column (SGE Analytical Science, Ringwood, Australia) and a flame ionization detector (FID). Helium was used as a carrier gas. The runtime was 36.3 min with an initial oven temperature of 100 °C, which was increased steadily to 220 °C (4.3 min to 170 °C, then 20 min to 200 °C and 12 min to 220 °C). The injector temperature was 280 °C and 1 μL was injected in split mode (50:1 split ratio). For identification and quantification of fatty acids, the C4–C24 FAME mixture (Supelco, St. Louis, USA) and C13:0 tridecanoic acid internal standard (Sigma-Aldrich, St Louis, USA) standards were used. Sample chromatograms can be found in Additional file 1: Figure S2.

### GC–MS fatty acid analysis

Identification and quantification of rare fatty acids, such as cis-vaccenic acid (C18:1n7) were performed by GC–MS. Analyses were carried out on an Agilent 6890 Series gas chromatograph (GC; Agilent, Wilmington, DE, USA) in combination with an Autospec Ultima mass spectrometer (MS; Micromass, Manchester, England) using an EI ion source. The GC was equipped with a CTC PAL Autosampler (CTC Analytics, Zwingen, Switzerland). Separation was carried out on a 60 m Restek column (Rtx-2330) with 0.25 mm I.D. and a 0.2 µm film thickness of fused silica 90% biscyanopropyl/10% cyanopropylphenyl polysiloxane stationary phase (Restek, Bellefonte, PA, USA). Helium was used as a carrier gas at 1.0 mL/min constant flow. The EI ion source was used in positive mode, producing 70 eV electrons at 250 °C. The MS was scanned in the range 40–600 m/z with 0.3 s scan time, 0.2 s inter scan delay, and 0.5 s cycle time. The transfer line temperature was set to 270 °C. The resolution was 1200. A split ratio of 1/10 was used with injections of 1.0 µL sample volume. Identification of fatty acids was performed by comparing retention times with standards as well as MS library searches. The MassLynx version 4.0 (Waters, Milford, MA, USA) and the NIST 2014 Mass Spectral Library (Gaithersburg, MD, USA) was used. The GC oven had a start temperature of 65 °C, which was held for 3 min, before the temperature was raised to 150 °C (40 °C/min), held for 13 min, and again increased to 151 °C (2 °C/min), held for 20 min, followed by a slow increase to 230 °C (2 °C/min), held for another 10 min, before finally increasing to 240 °C (50 °C/min), which was held for 3.7 min.

### HPLC glucose analysis

Glucose was quantified using an UltiMate 3000 UHPLC system (Thermo Scientific, Waltham, USA) equipped with RFQ-Fast Acid H + 8% (100 × 7.8 mm) column (Phenomenex, Torrance, USA) and coupled to a refractive index (RI) detector. Samples were diluted ten times before analysis, then filter sterilized and subsequently eluted isocratically at 1.0 mL min^−1^ flow rate in 6 min with 5 mM H_2_SO_4_ mobile phase at 85 °C column temperature.

### Data analysis

FTIR spectra (4000–500 cm^−1^) were preprocessed by transforming to 2nd derivative form with the Savitzky–Golay (S–G) method (2nd degree polynomial, windows size 15), followed by Extended Multiplicative Scatter Correction (EMSC) with linear and quadratic components [26]. Principal component analysis (PCA) of the EMSC corrected FTIR data and auto-scaled GC fatty acid data was performed in The Unscrambler X, V10.5 (CAMO, Oslo, Norway). Partial Least Square Regression (PLSR) between FTIR data (S–G and EMSC) and GC fatty acid data was performed with a leave-one-biological-replicate-out cross validation scheme, and with limiting the maximum number of PLS factors to ten.

## Results

### Diversity of macro- and microscopic morphology of Mucoromycota fungi grown in the Duetz-MTPS

**Fig. 1:**
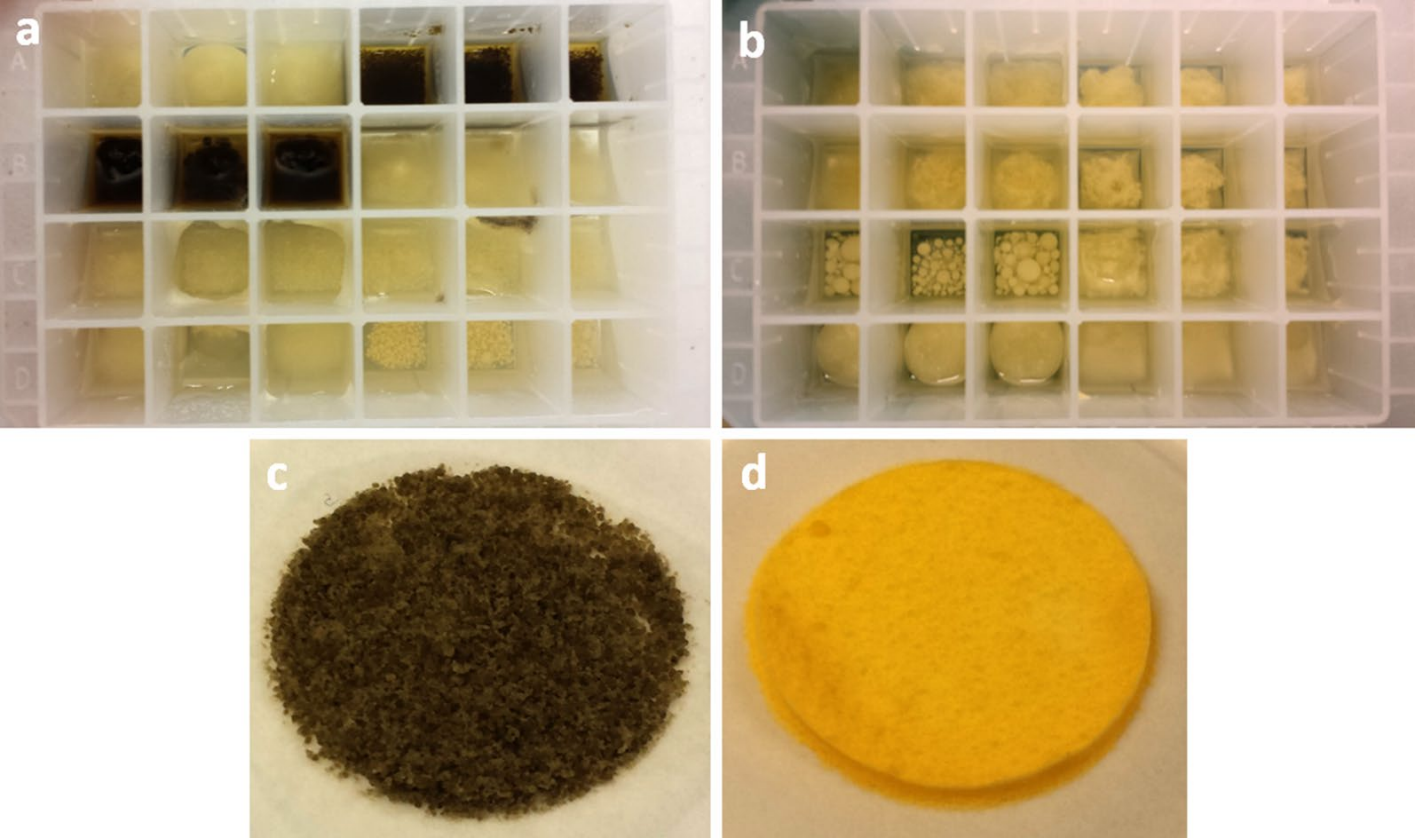
**a**, **b** Variety of Mucoromycota fungi morphologies grown under lipid accumulation conditions in Duetz-MTPS (small-big pellets, dispersed, wall-growth), **c** *Mucor mucedo* UBOCC-A-101353, **d** *Mucor hiemalis* UBOCC-A-101359

**Fig. 2:**
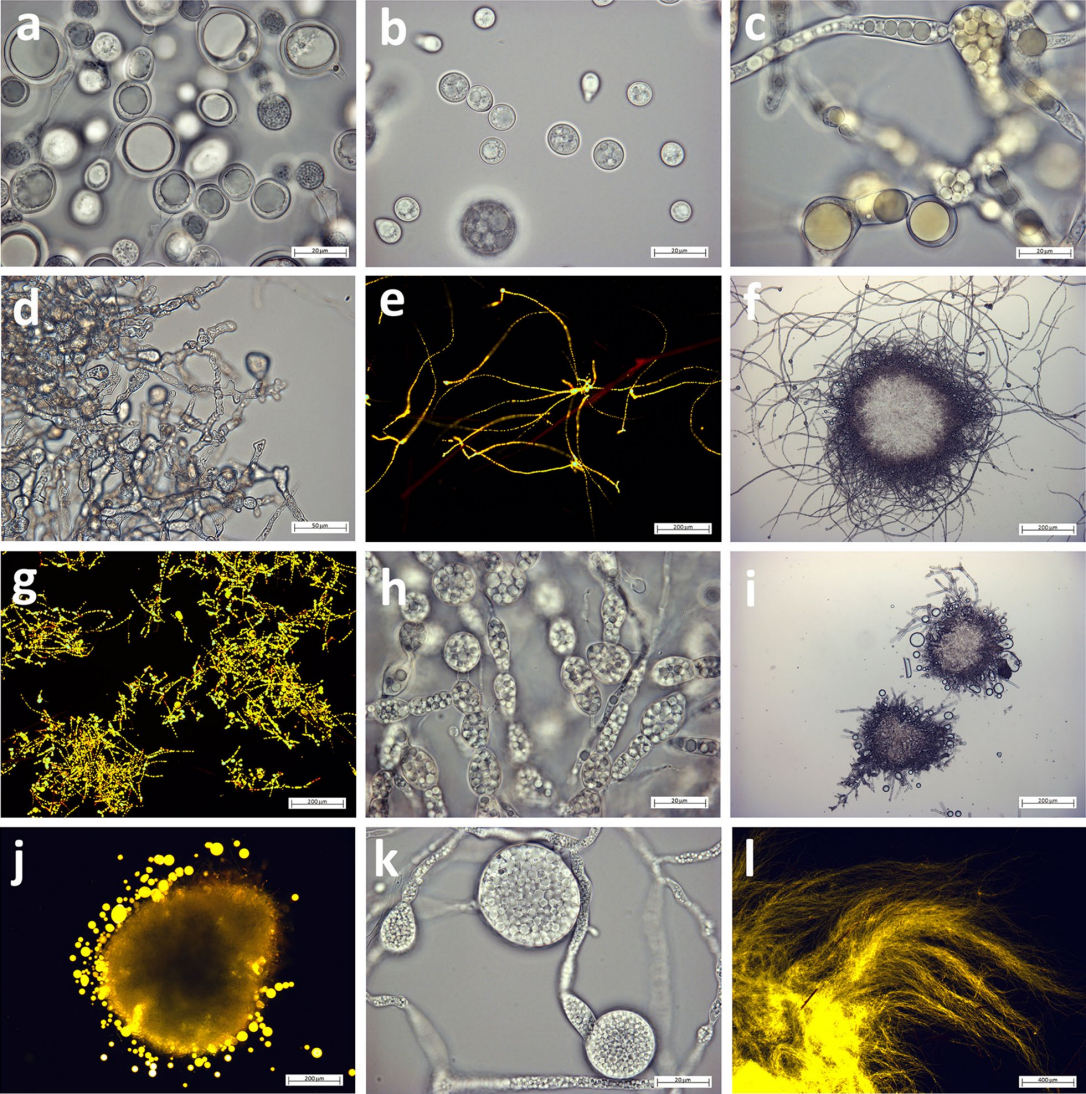
Different microscopic morphologies of oleaginous mycelium of Mucoromycota fungi. **a** *Mucor racemosus* FRR 3336, **b** *Mucor circinelloides* CCM 8328 (single cell form), **c** *Mucor hiemalis* UBOCC-A-101359, **d** *Rhizopus oryzae* CCM 8075, **e** *Umbelopsis isabellina* UBOCC-A-101350, **f** *Umbelopsis ramanniana* CCM F-622, **g** *Umbelopsis vinacea* UBOCC-A-101347, **h** *Umbelopsis vinacea* CCM F-539, **i** *Absidia coerulea* CCM 8230, **j** *Cunninghamella blakesleeana* VKM F-993, **k** *Mortierella zonata* UBOCC-A-101348, **l** *Mortierella hyalina* VKM F-1854

A variety of macroscopic structures were observed during the cultivation of Mucoromycota fungi under lipid accumulation conditions in the Duetz-MTPS (Fig. 1a, b). Forty-nine strains, mainly from *Mucor* and *Rhizopus* genera, grew in a dispersed hyphal form, forty-two strains from genera *Umbelopsis*, *Absidia*, *Cunninghamella*, *Lichtheimia* and *Mortierella* grew in the form of pellets with different size, while the remaining strains showed mixed macroscopic morphology. Wall growth was observed for several strains (especially in *Mucor*, *Rhizopus* and *Mortierella* genera, because dispersed mycelium and fluffy pellets were more prone to attach to the wall than globular pellets), which resulted in a more pronounced sporulation. Most of the fungal biomass had a white color with the exception of some *Mucor* strains which had pale yellow (*M. circinelloides* FRR 5020, FRR 5021, FRR 4846, *M. mucedo* UBOCC-A-101361), intense yellow (*M. hiemalis* UBOCC-A-101359, 101360, 111119, 112185) or dark green color (*M. mucedo* UBOCC-A-101353, 101362), due to the production of carotenoids and other pigments (Fig. 1c, d). All studied Mucoromycota fungi grew in a filamentous form, except in the case of certain *Mucor* spp., for which both filamentous and single cell yeast-like forms were observed (Fig. 2b). Lipid bodies (LBs) of *Mucor* spp. reached in some cases 20 μm in diameter (Fig. 2a). *M. hiemalis* strains showed yellow-colored LBs due to the presence of lipophilic carotenoids (Fig. 2c). Strains of *Rhizopus* spp. displayed branched mycelium with a limited amount of LBs (Fig. 2d). Hyphae of *Umbelopsis*, *Cunninghamella*, *Lichtheimia* and *Mortierella* were filled with 2–5 μm LBs (Fig. 2e–l). The mycelium of *Mortierella zonata* UBOCC-A-101348 had swollen hyphal tips, which were completely filled with LBs (Fig. 2k). Extracellular LBs were observed for fungi with high lipid content (*Absidia*, *Umbelopsis* and *Cunninghamella*) probably resulting from sample preparation (Fig. 2i, j). Yellow-gold fluorescence of the Nile-red stained samples confirmed the presence of neutral lipids in intra- and extracellular LBs (Fig. 2e, g, j, l).

### Biomass concentration and lipid content of Mucoromycota fungi

**Fig. 3:**
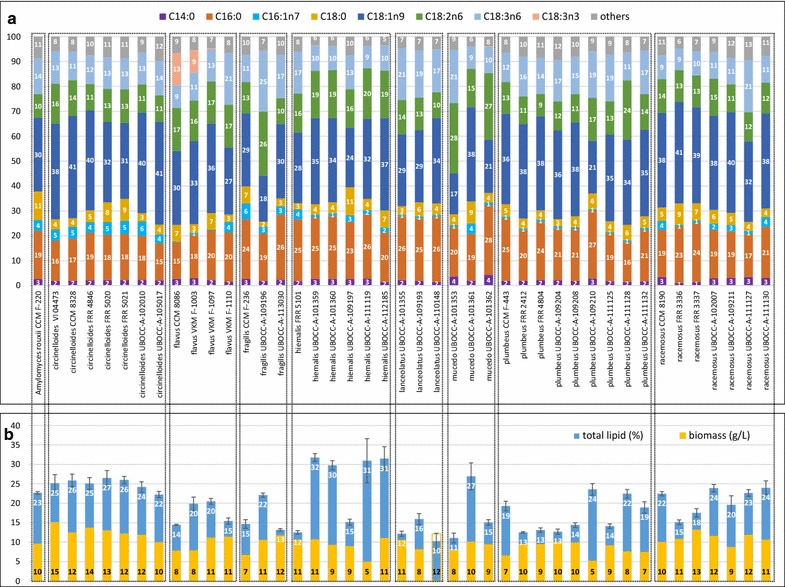
**a** Fatty acid profile (%), **b** total lipid content of biomass (%) and biomass concentration (g/L) of *Amylomyces rouxii* and *Mucor* fungi

**Fig. 4:**
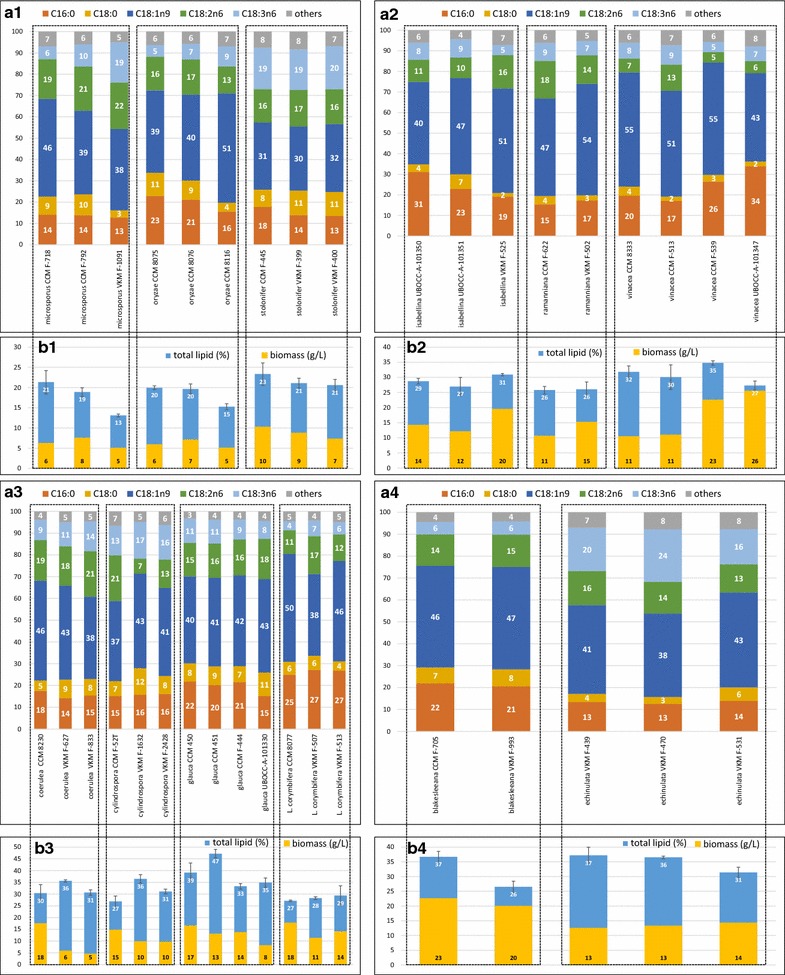
**a** Fatty acid profile (%), **b** total lipid content of biomass (%) and biomass concentration (g/L) of *Rhizopus* (1), *Umbelopsis* (2), *Absidia/Lichtheimia* (3), *Cunninghamella* (4) fungi

**Fig. 5:**
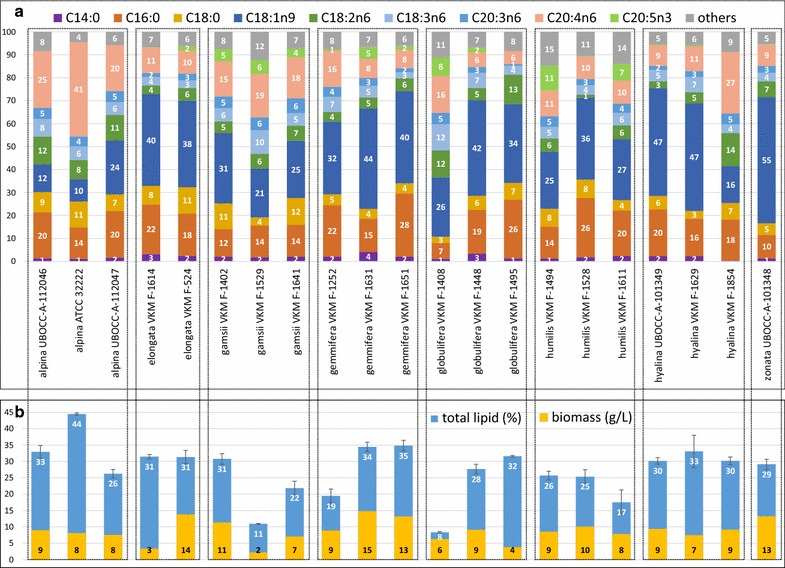
**a** Fatty acid profile (%), **b** total lipid content of biomass (%) and biomass concentration (g/L) of *Mortierella* fungi

**Fig. 6:**
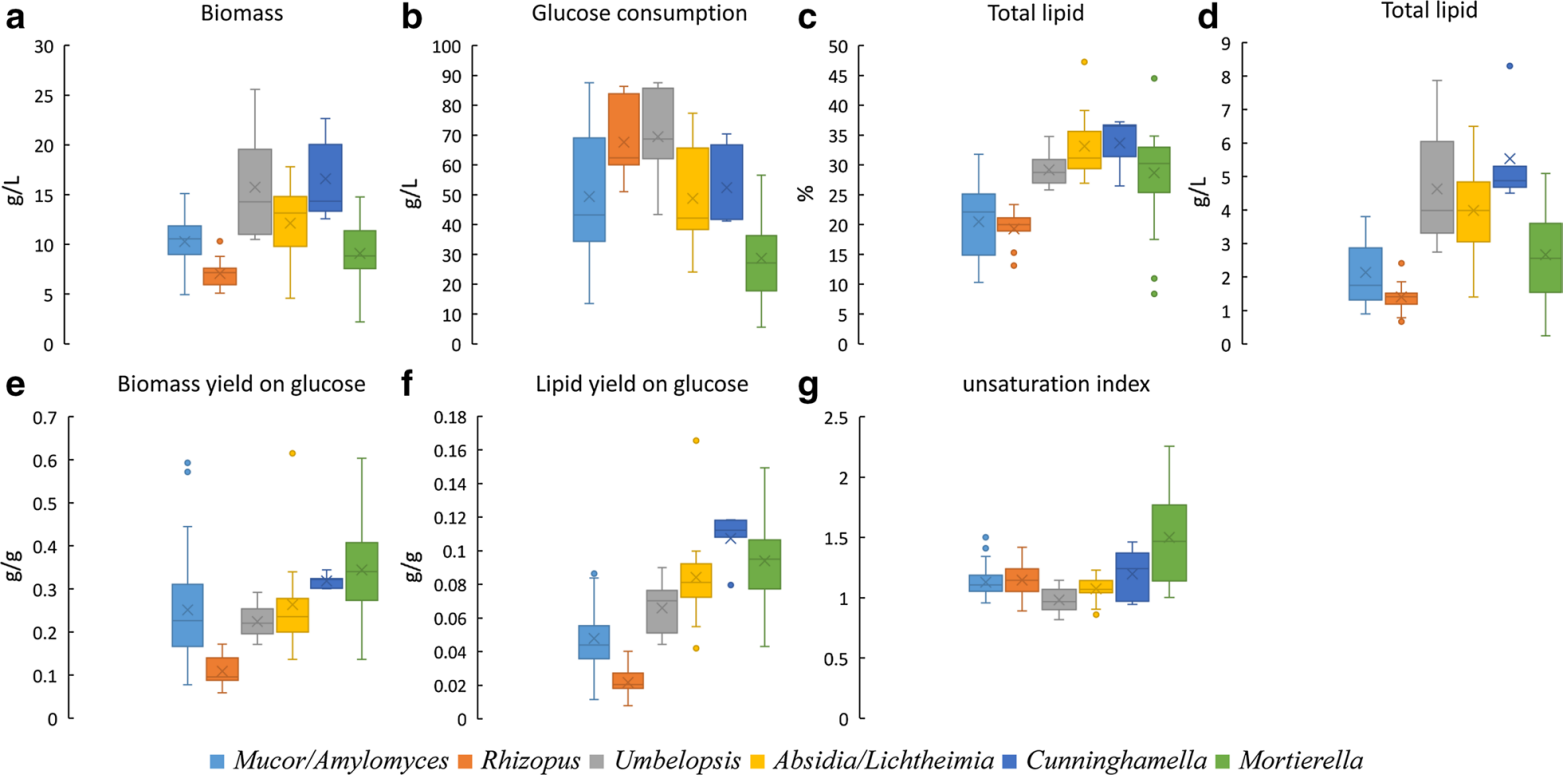
Main fermentation parameters for the tested Mucoromycota genera. **a** Biomass concentration (g/L), **b** glucose consumption (g/L), **c** total lipid content of biomass (%), **d** lipid concentration (g/L medium), **e** biomass- and **f** lipid yield on glucose (g/g), **g** unsaturation indices (−)

The (submerged) biomass concentration and total lipid content of each tested strain are reported in Fig. 3b (*Mucor* strains and *Amylomyces rouxii*), Fig. 4b1–b4 (*Rhizopus*, *Umbelopsis*, *Absidia*, *Lichtheimia* and *Cunninghamella*) and Fig. 5b (*Mortierella*). The best ten oleaginous Mucoromycota fungi according to biomass concentration, total lipid content in biomass and total lipid concentration can be seen in Additional file 1: Table S1. The summary of the results is presented for each genus in Fig. 6a, c.

*Umbelopsis* (min. 11–max. 26 g/L, average 15.7 g/L) and *Cunninghamella* (13–23 g/L, average 16.6 g/L) strains reached the highest biomass concentration with *Cunninghamella blakesleeana* CCM-705, *Umbelopsis vinacea* CCM F-539, and *U. vinacea* UBOCC-A-101347 showing the highest biomass, ranging from 22.6 to 25.6 g/L. Fungi from the other Mucoromycota genera, showed typically lower biomass concentration, in the range of 2–18 g/L. *Rhizopus* strains grew poorly (5–10 g/L, average 7.1 g/L) despite of their high glucose consumption (average 68 g/L) (Fig. 6b). It is worth mentioning that *Rhizopus* spp. acidified the growth medium, indicating acid production, which may have negatively affected their growth. In general, *Mortierella* spp. grew slowly in the Duetz-MTPS and several strains did not grow properly in the standard conditions (90 g/L glucose, 28 °C), therefore, glucose concentration and temperature had to be lowered (Table 1). *M. globulifera* VKM F-1408 (2 g/L), VKM F-1448 (6 g/L) and *M. gamsii* VKM F-1529 (9 g/L) did not grow in the Duetz-MTPS, and reached low biomass concentration in SFs as well. In *Mucor* genus, the biomass concentration was the highest in *M. circinelloides* species: five strains reached 12–15 g/L.

All studied strains of *Umbelopsis*, *Absidia*, *Lichtheimia* and *Cunninghamella* spp. could be considered as oleaginous as they had a total lipid content ranging from 26 to 47%. *Absidia* strains, except *A. cylindrospora* CMM F-52T, accumulated more than 30% of lipids and the highest lipid content among all tested fungi, was achieved in *Absidia glauca* CCM 451 with 47.2 ± 1.8% of total lipid content. Among *Umbelopsis* and *Cunninghamella* strains, the highest lipid content was between 35 and 37% in *U. vinacea* CCM F-539, *C. blakesleeana* CMM F-705, *C. echinulata* VKM F-439 and *C. echinulata* VKM F-470. The lipid content in *Mucor* spp. varied between 10 and 32%, showing large intraspecies diversity as well (e.g. 12% in *M. hiemalis* FRR 5101 and 32% in *M. hiemalis* UBOCC-A-101359). In the genus *Mucor*, the best lipid producers were found within *M. hiemalis*, where four strains reached 30–32% of lipid content. All *M. circinelloides* strains were oleaginous with a lipid content of 22–27%. The lipid content of *Rhizopus* spp. was moderate, with highest value of 23% in *Rhizopus stolonifer* CCM F-445. Most *Mortierella* strains were oleaginous and half of them reached more than 30% lipid content in their biomass. *M. alpina* ATCC 32222 had the second highest lipid content from all tested fungi (44.5 ± 0.3%).

### Fatty acid profiles of Mucoromycota fungi

**Fig. 7:**
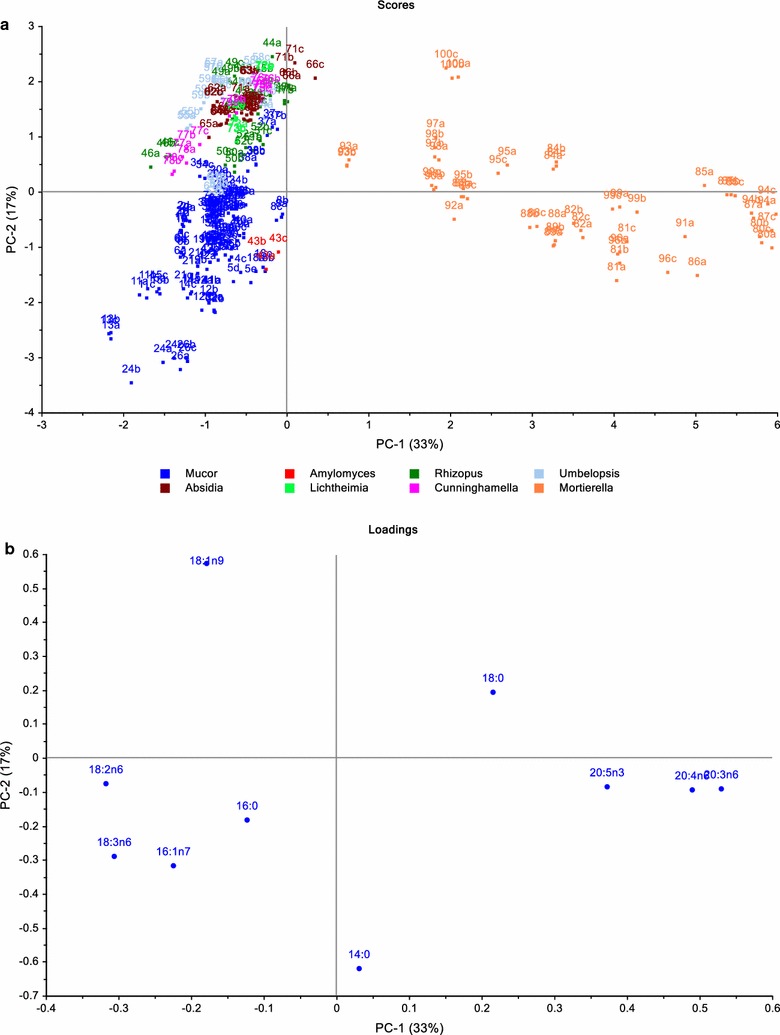
**a** Scores plot of GC fatty acid data. Numbers in the scores plot refer to strains in Table 1, while letters refer to biological replicates (3 biological replicates: a, b, c or 5 biological replicates: a, b, c, d, e for *M. circinelloides* strains). **b** Loadings plot of GC fatty acid data. Fatty acid data was autoscaled before PCA

The FA profiles of the tested strains were analyzed by PCA (the most important FA only). PCA score and loading plots are shown in Fig. 7a, b. PC1 separates *Mortierella* strains from those of the Mucorales order primarily based on the presence or absence of C20 polyunsaturated FAs (DGLA, ARA and EPA). PC2 separates Mucorales order into two clusters. *Mucor* and *Amylomyces* genera are characterized by high myristic acid (C14:0), palmitoleic acid (C16:1n7) and GLA content, while *Rhizopus*, *Umbelopsis*, *Absidia*, and *Lichtheimia*, *Cunninghamella* genera are generally characterized by high oleic acid (C18:1n9, OA) content. The detailed fatty acid profile of all tested Mucoromycota fungi can be found in Additional file 2.

### Production of high-value PUFA in Mucoromycota fungi

Main fatty acid profiles of *Mucor* and *Amylomyces rouxii* can be seen in Fig. 3a, while those of *Rhizopus*, *Umbelopsis*, *Absidia*, *Lichtheimia*, *Cunninghamella,* and *Mortierella* are shown in Fig. 4a1–a4 and 5a, respectively. The 10 strains showing the highest GLA and ARA production are presented in Additional file 1: Table S1.

In *Mucor* spp., the most abundant FA was OA, except in *M. mucedo* UBOCC-A-101362, 101353 and *M. fragilis* UBOCC-A-109196 for which either linoleic acid (C18:2n6, LA), or both LA and GLA content was higher than OA. Among all studied Mucoromycota fungi, *M. fragilis* UBOCC-A-109196 produced the highest percentage of GLA in the oil (24.5 ± 0.3%). *M. flavus* VKM F-1110 and *M. racemosus* UBOCC-A-111127 strains also produced more than 20% GLA, but only the latter one was oleaginous (23% total lipid content). Two *M. flavus* strains, CCM 8086 and VKM F-1003, also produced, in addition to 9.1–11.1% GLA, 13.0 and 9.0% α-linolenic acid (C18:3n3, ALA) in the oil, respectively (Additional file 1: Figure S2). Both strains were grown at low temperatures (15 and 20 °C) that likely increased the activity of ∆15-desaturase enzyme (ω3 desaturase), resulting in α-linolenic acid (C18:3n3, ALA) production. ALA was further desaturated by ∆6-desaturase leading to the 3.0–1.8% stearidonic acid (C18:4n3, SDA) and elongated to 0.5–0.9% eicosatrienoic acid (C20:3n3, ETE) (Additional file 1: Figure S3). Interestingly, the expression of ∆15-desaturase enzyme was much weaker in *M. flavus* VKM-1097 grown at 20 °C, where only 0.4% ALA was produced along with 1.3% SDA and no ETE detected, while in *M. racemosus* UBOCC-A 111127 the low cultivation temperature (15 °C) did not lead to ALA, SDA or ETE production. In *Rhizopus* strains, the GLA content varied between 5.5 and 20.3% in the oil. *R. stolonifer* strains produced the highest amount of GLA (19.0–20.3%), while its content varied greatly in *R. microsporus* (6.0–18.8%), and the lowest content of GLA in fungal oil was achieved in *R. oryzae* strains (5.5–9.4%). GLA content in oil was low in *Umbelopsis* strains, varying between 4.9 and 9.4%. Concerning *Absidia* and *Lichtheimia* spp., GLA content was the lowest in *L. corymbifera* strains (4.1–7.0%) and the highest in *A. cylindrospora* strains (13.5–16.9%). Within members of the *Cunninghamella* genus, *C. echinulata* strains produced much higher level of GLA (16.0–24.0%) than *C. blakesleeana* strains (5.6–6.1%). *C. echinulata* VKM F-470 showed the second highest GLA content in the oil from all tested strains with a level of 24.0 ± 1.1%.

*Mortierella* strains produced significant amounts of C20 PUFAs, mainly DGLA, ARA and EPA. The average unsaturation index (calculated based on Suutari et al. [27]) was also higher in this genus (1.50 combined and 1.43 for 28 °C cultivation only) than in the other genera (0.98–1.20) (Fig. 6g). The *Mortierella* strains, which were cultivated at 15 °C, produced higher content of omega-3 FAs than at 28 °C, indicating the increased activity of ω3-desaturase (∆15, ∆17) enzymes [28]. Comparing the fungal oil of *Mortierella* spp. at low (15 °C) and high (28 °C) cultivation temperatures, the ALA content was on average 0.53% (max. 0.8%) and 0.08%, while the SDA content was 0.9% (max. 1.4%) and 0.1%. The eicosatetraenoic acid (C20:4n3, ETA) content was 1.2% (max. 2.1%) and 0.08%, while EPA was found to be 6.6 (max. 10.8%) and 0.5%, respectively. In some species that were cultivated at 28 °C, ~ 2% EPA was found in the oil (*M. elongata* VKM-F524 and *M. globulifera* VKM F-1448), indicating a lower activity of ω3-desaturase at room temperature. DGLA was found in the oil the highest percentage in *M. gamsii* strains grown at 15 °C, with values ranging from 5.1 to 6.5%. The industrially relevant *M. alpina* ATCC 32222 (28 °C) strain produced the highest content of ARA in the oil (41.1 ± 0.8%, unsaturation index: 2.25), followed by *M. hyalina* VKM F-1854 (26.7 ± 1.2%) and *M. alpina* UBOCC-A-112046 (24.6 ± 1.2%). *M. globulifera* VKM F-1408 (15 °C) produced various PUFA at high levels (unsaturation index: 2.16): GLA 11.5 ± 1.1%, DGLA 4.9 ± 0.1%, ARA 16.1 ± 0.6%, EPA 8.0 ± 1.1%. The highest EPA content in oil was achieved in *M. humilis* VKM F-1494 (15 °C): 10.8 ± 0.3% (Additional file 1 Figure S2).

In addition to the above described FAs, Mucoromycota fungi also produced odd chain FAs in smaller quantities, amongst others: pentadecylic acid (C15:0, average 0.3%, max. 1.5%), margaric acid (C17:0, average 0.6%, max 3.0%), heptadecenoic acid (C17:1n7, average 0.3%, max. 1.3%). The *cis*-vaccenic acid (C18:1n7, average 0.3%, max. 1.3%) was observed in most fungi. Furthermore, lignoceric acid (C24:0, average 0.8%, max. 3.0%) and nervonic acid (C24:1n9 average 0.2%, max. 1.8%) were also common in the fungal oil. From the trans FAs, the fatty acid C18:2n9t occurred most frequently and in highest amount (average 0.5%, max. 2.4%).

### Low-value fatty acids in Mucoromycota fungi for biodiesel production

The tested strains were also evaluated regarding their possible use for biodiesel production. The two most important properties of FAs that affect the fuel properties are the carbon chain length and the number of double bonds [29]. The ideal fatty acid composition for good oxidative stability of biodiesel is a ratio of C16:1, C18:1, C14:0 fatty acid 5:4:1 [30, 31]. The EN14214 standard for biodiesel describes the required specifications of biodiesel (FAME): amongst other criteria, the cetane number (CN) should be higher than 51 (the higher the better), the density at 15 °C should be between 860 and 900 kg m^−3^, the iodine value (IV, g I_2_/100 g) should be less than 120, the GLA content should be less than 12%, and the PUFA content with four or more double bonds less than 1%. In the present study, CN, density, IV and the higher heating value (HHV, MJ kg^−3^) biodiesel properties were calculated from FA composition, according to Ramírez-Verduzco et al. [31]. These values for all tested strains can be found in Additional file 2.

Based on these calculations, forty-two strains met the requirement of EN14214 standard: 17 *Mucor* strains, 5 *Rhizopus*, all *Umbelopsis*, 6 *Absidia*, all *Lichtheimia* and 2 *Cunninghamella.* Strains with high ALA/GLA and C20 PUFA content (e.g. *Mucor* spp. with more than 12% GLA, *R. stolonifer*, *A. cylindrospora*, *C. echinulata* and *Mortierella* spp.) were not suitable for biodiesel production. The ten best biodiesel producers based on their total lipid content of biomass, lipid concentration and cetane number can be seen in Additional file 1: Table S2. *U. vinacae* CCM F-539 and UBOCC-A-101347 had the best biodiesel characteristics based on the highest CN value (62.8–62.3), lowest iodine value (70.6–71.7), and amongst the highest HHV values (39.75–39.81 MJ kg^−1^).

### FTIR spectroscopy

**Fig. 8:**
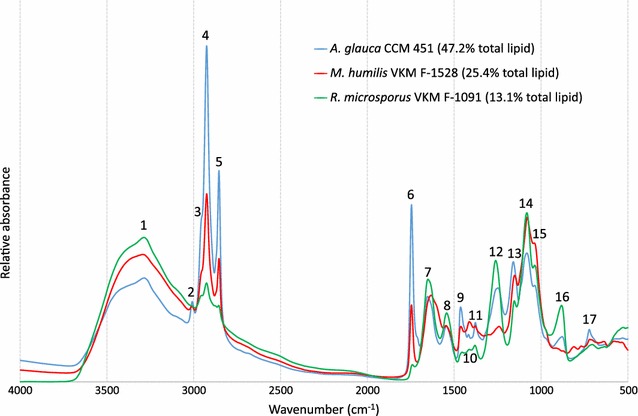
(EMSC corrected) FTIR spectra of Mucoromycota fungi with low, intermediate, and high total lipid content. Peak assignments can be found in Additional file 1: Table S3

Fungal biomass was also measured by high-throughput FTIR spectroscopy (Additional file 3) as a rapid method for the screening of Mucoromycota fungi for single cell oil production. FTIR spectra of three Mucoromycota fungi with very different lipid content can be seen in Fig. 8. The most important peaks are assigned in Additional file 1: Table S3. We observe that the lipid related FTIR peaks (No. 2–6, 9, 11, 13, 17) change according to the lipid content of the fungi (measured by GC-FID).

**Fig. 9:**
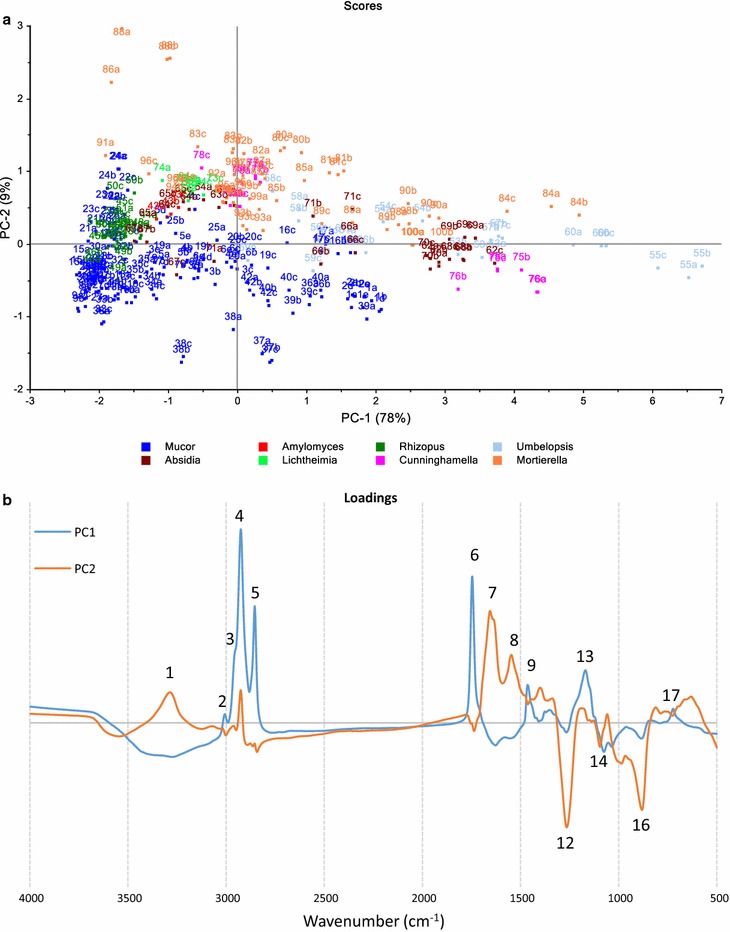
**a** Scores and **b** loadings (PC1-2) plots of FTIR data (EMSC corrected). The explained variances for the first five PCs are 78, 9, 5, 3 and 2%. Numbers in the scores plot refers to strains in Table 1, while letters refer to biological replicates (3 biological replicates: a, b, c or 5 biological replicates: a, b, c, d, e for *M. circinelloides* strains). Peak assignments can be found in Additional file 1: Table S3

A PCA analysis of the EMSC corrected FTIR spectra for the spectral region 4000–500 cm^−1^ is shown in Fig. 9. Biological replicates (labelled by a–c or a–e) are located close to each other in the score plot confirming good cultivation reproducibility in the Duetz-MTPS. The main separation of fungi is based on lipid content of the biomass (PC1, 78% variance) demonstrated by lipid specific peaks in the loading plot. PC2 explains 9% of the variance. The ratio of protein (7, 8) and phosphate (12, 14, 16) is responsible for the separation of strains in PC2. *Mucor* species have predominantly negative PC2 scores, which can be explained by their very high polyphospate content in their cell wall [32]. The FTIR data indicated that *Mucor*/*Amylomyces* and *Rhizopus* have lower total lipid content on average than *Absidia*, *Umbelopsis*, *Cunninghamella* and *Mortierella* genera, which is in accordance with the GC measurement results (see also Fig. 6c).

FTIR spectra of Mucoromycota strains were used to estimate the lipid content in the mycelium (measured by GC-FID analysis). Univariate methods were tested for the whole set of studied strains and individually for each genus, and were compared to the multivariate method (PLSR) [23]. The univariate methods were based on the height of C=O ester peak (1745 cm^−1^) and the ratio of C=O ester and amide I (1655 cm^−1^) peak heights [33, 34]. The results of these analyses can be seen in Additional file 1: Table S4. Univariate regression results are only acceptable in case of *Mucor*/*Amylomyces*, *Rhizopus*, *Absidia*/*Lichtheimia* and *Mortierella* genera, and were clearly outperformed by the PLSR method.

## Discussion

It is known that reproducible cultivation of filamentous fungi is a challenging task due to their varying morphology and adherent wall growth [21, 35]. Moreover, in many previous studies focusing on the screening of lipid production in SF cultures [3, 16, 17], biological replicates were not made, either due to time (cultivation, extraction) and/or space (shaker) limitations, making the reproducibility of experiments difficult to judge. The Duetz-MPTS enabled good reproducibility of biological replicate cultures. Indeed, the pooled variation coefficients of biological replicates (average of all data) for total lipid content, biomass concentration, and consumed glucose were 6.1, 12.1 and 5.5%, respectively. Thus, the variability between biological replicates was very low, even if spores originated from distinct pre-cultures, and spore inocula derived from each pre-culture were not standardized at the same concentration. In our previous study, the good reproducibility of this cultivation method was also demonstrated for oleaginous filamentous fungi [23]. Similarly, other studies have shown that microtiter plate cultivation can offer very good (sometimes better) reproducibility for filamentous fungi, bacteria [36–38] and yeast [39] than SF based cultivation. Nevertheless, wall growth was an issue in the current study, especially with fungi with dispersed mycelium or fluffy pellet morphology (mainly *Mucor*, *Rhizopus* and *Mortierella* spp.). Wall grown biomass weight can exceed the weight of the submerged biomass weight (data not shown). In the current study, the wall-grown biomass was not collected, therefore, the reported biomass concentration should be considered as the submerged biomass concentration or a ‘minimum’ value. In some cases the reported biomass values are, therefore, severely underestimated, affecting also other reported fermentation parameters (e.g. total lipid; g/L, yield values; g/g) (Fig. 6d–f, Additional file 1: Table S1, S2). To solve wall growth of filamentous organisms in MTP, addition of glass beads or carboxypolymethylene to the medium, and mutation to pellet morphology have been successfully applied [37, 38, 40, 41].

Reproducibility of total lipid content measurement (wt%) was estimated by performing three times the extraction- transesterification—GC-FID procedure on a *Mucor flavus* CCM 8086 and *Absidia glauca* CCM 451 biomass samples (i.e. three technical replicates). The variation coefficient of total lipid content was very low for both samples (0.9 and 5.3%, respectively), indicating the reliability of the developed procedure (Additional file 2).

In the present study, we confirmed the potential of several species previously known for high value PUFA (i.e. *Mucor* spp., *Cunninghamella echinulata*, *Rhizopus stolonifer, Mortierella alpina*) and biodiesel production (*Umbelopsis* spp., *Cunninghamella blakesleeana* etc.) [7, 14, 15, 19, 42, 43]. Since Duetz-MTPS offers much higher throughput (enabling to run sufficient amount of replicates) than SF cultures and requires lower space and less medium, our method appears as the most suitable one for screening experiments. In addition, we found much higher total lipid content of biomass (27% vs. 13% on average) and high-value PUFA content of oil (e.g. in *M. gemmifera* VKM F-1252 we found 4.3% DGLA vs. 0 and 15.9% ARA vs. 10.3%) in eleven *Mortierella* strains (VKM F-525, F-1611, F-1408, F-1448, F-1495, F-1631, F-1252, F-524, F-1614, F-1402, F-1529) that were previously screened by Eroshin et al. [14] in an agar-based medium. These differences can be explained by the fact that different cultivation mode and medium composition were used in the present study, i.e. submerged cultures in a medium with a high carbon-to-nitrogen ratio, allowing to reach a higher lipid content in the tested fungi.

*Absidia* species have rarely been mentioned in the literature as oleaginous fungi. According to our results, these species deserve more attention as they appeared as excellent lipid producers. To our best knowledge, the only work which is focused on *Absidia* spp. lipid production is that from Puttalingamma [44]. In this study, 11 *Absidia/Lichtheimia* strains were screened in media containing different carbon sources. High biomass and total lipid yields were obtained with up to 43.6 g/L with *L. corymbifera* MTCC 1549 and 51.4% in *A. repenses* MTCC 1327. However, in Puttalingamma’s study, only gravimetric lipid yield was reported. It should be stressed that the determination of gravimetric lipid yield can lead to severe overestimation of the fatty acid-based lipid content (i.e. tri, di- and monoglycerides, glycerophospholipids, and free fatty acids) [45], and is not as reliable method as GC-FID quantification of FAME using an internal standard. Another benefit of the transesterification of FAs to FAMEs is that it represents directly the biodiesel potential. Moreover, in the present study, detailed fatty acid profiles of 13 *Absidia*/*Lichtheimia* strains were obtained in contrast to the work of Puttalingamma [44].

Another interesting finding of the present study was the unusual concomitant production of comparable amount of α-linolenic acid and γ-linolenic acid in *M. flavus* CCM 8086 and VKM F-1003 after cultivation at 15 and 20 °C, respectively. It is well known that cold temperature stimulate the expression of ω3 desaturase enzymes in fungi, leading to omega-3 fatty acid production [15, 16, 28, 46]. This phenomenon was also observed for *Mortierella* spp., for which EPA production increased at 15 °C as compared to 28 °C. Nonetheless, to our best knowledge concomitant GLA and ALA production has not yet been reported in *Mucor* spp.

Finally, we showed that FTIR spectroscopy can be applied as a rapid analytical method for the prediction of total lipid content in the biomass using multivariate regression. In addition, FTIR spectroscopy is a well-established high-throughput method for the classification of microorganisms, due to its ability to provide highly reproducible spectral fingerprints [47]. Moreover, it can be expected that FTIR spectroscopy can be used for the prediction of fatty acid composition as well, as demonstrated previously [23]. These aspects will be investigated in a follow-up article.

## Conclusions

This study showed that the Duetz-MTPS is suitable for the reproducible cultivation of a large variety of Mucoromycota fungi, while revealing details about their lipid production potential. Using this method, we found several promising candidates for PUFA and biodiesel production. The benefits of this technique are the very high throughput (plates can be stacked in a shaker) and the possibility to automate the system. Further development currently undertaken in our laboratory includes the use of a robotic system allowing biomass-liquid separation and biomass washing, homogenization and pipetting on silicone plates, prior to HTS–FTIR analysis [48]. This fully automated high-throughput cultivation-analytical platform may allow an even more efficient screening of microbial bioprocesses in the future. Wall-growth of fungi can hinder automation of the system, therefore, it should be prevented in the future. Furthermore, we showed the potential of high-throughput FTIR spectroscopy, as a rapid analytical method for the detection of high lipid producers, before performing the detailed fatty acid analysis by gas chromatography.

## Additional files

**Additional file 1.** Tables and Figures.

**Additional file 2.** GC-FID and GC-MS fatty acid analysis results.

**Additional file 3.** FTIR spectra of fungal biomass.
