# Supplementary material for: High-throughput screening of Mucoromycota fungi for production of low- and high-value lipids
Source: Biotechnol Biofuels. 2018 Mar 14;11:66. doi: 10.1186/s13068-018-1070-7 (PMC5851148; doi:10.1186/s13068-018-1070-7)
Supplement: Supplementary file 1 — Additional file 1. Tables and Figures. [file 13068_2018_1070_MOESM1_ESM.pdf]

Additional file 1

High-throughput screening of Mucoromycota fungi for production of low- and high-value lipids

Gergely Kosa[1*] (gergely.kosa@nmbu.no), Boris Zimmermann[1] (boris.zimmermann@nmbu.no), Achim Kohler[1] (achim.kohler@nmbu.no), Dag Ekeberg[2] (dag.ekeberg@nmbu.no), Nils Kristian Afseth[3] (nils.kristian.afseth@nofima.no), Jerome Mounier[4] (jerome.mounier@univ-brest.fr), Volha Shapaval[1] (volha.shapaval@nmbu.no)

(a) Faculty of Science and Technology, Norwegian University of Life Sciences, P.O. Box 5003, 1432 Ås, Norway

(2) Faculty of Chemistry, Biotechnology and Food Science, Norwegian University of Life Sciences, P.O. Box 5003, 1432 Ås, Norway

(3) Nofima AS, Osloveien 1, 1433 Ås, Norway

(4) Université de Brest, EA3882 Laboratoire Universitaire de Biodiversité et Ecologie Microbienne, IBSAM, ESIAB, Technopôle Brest Iroise, 29280 Plouzané, France

Correspondence address: Faculty of Science and Technology, Norwegian University of Life Sciences, Postbox 5003, 1432 Ås, Norway

Table of contents: Page

The ten best strains 2

Peak assignment in FTIR spectra of filamentous fungi 3

Prediction of total lipid content from FTIR spectra 4

Classification of Mucoromycota fungi 5

GC-FID chromatogram examples 6

Pathways for the formation of PUFA in microorganisms 7

References 8

Table S1 Mucoromycota strains showing the 10 highest biomass concentration (g/L), total lipid content of the biomass (%), total lipid concentration (g/L medium), GLA and ARA production (% of total fatty acids, g/L medium)

	Biomass (g/L)		Total lipid (%)		Total lipid (g/L)
Umbelopsis vinacea UBOCC-A-101347	25.6	Absidia glauca CCM 451	47.2	Cunninghamella blakesleeana CCM F-705	8.3
Cunninghamella blakesleeana CCM F-705	22.6	Mortierella alpina ATCC 32222	44.5	Umbelopsis vinacea CCM F-539	7.9
Umbelopsis vinacea CCM F-539	22.6	Absidia glauca CCM 450	39.1	Umbelopsis vinacea UBOCC-A-101347	7.0
Cunninghamella blakesleeana VKM F-993	20.0	Cunninghamella echinulata VKM F-439	37.2	Absidia glauca CCM 450	6.5
Umbelopsis isabellina VKM F-525	19.6	Cunninghamella blakesleeana CCM F-705	36.7	Absidia glauca CCM 451	6.2
Lichtheimia corymbifera CCM 8077	17.8	Cunninghamella echinulata VKM F-470	36.5	Umbelopsis isabellina VKM F-525	6.0
Absidia coerulea CCM 8230	17.5	Absidia cylindrospora VKM F-1632	36.5	Absidia coerulea CCM 8230	5.3
Absidia glauca CCM 450	16.6	Absidia coerulea VKM F-627	35.6	Cunninghamella blakesleeana VKM F-993	5.3
Umbelopsis ramanniana VKM F-502	15.3	Absidia glauca UBOCC-A-101330	34.8	Mortierella gemmifera VKM F-1631	5.1
Mucor circinelloides VI 04473	15.1	Mortierella gemmifera VKM F-1651	34.8	Cunninghamella echinulata VKM F-470	4.9

	GLA (% total FAs)		ARA (% total FAs)
Mucor fragilis UBOCC-A-109196	24.5	Mortierella alpina ATCC 32222	41.1
Cunninghamella echinulata VKM F-470	24.0	Mortierella hyalina VKM F-1854	26.7
Mucor mucedo UBOCC-A-101353	21.5	Mortierella alpina UBOCC-A-112046	24.6
Mucor racemosus UBOCC-A-111127	20.9	Mortierella alpina UBOCC-A-112047	20.0
Mucor flavus VKM F-1110	20.9	Mortierella gamsii VKM F-1529	18.9
Mucor lanceolatus UBOCC-A-101355	20.9	Mortierella gamsii VKM F-1641	17.9
Rhizopus stolonifer VKM F-400	20.3	Mortierella globulifera VKM F-1408	16.1
Cunninghamella echinulata VKM F-439	19.7	Mortierella gemmifera VKM F-1252	15.9
Rhizopus stolonifer CCM F-445	19.4	Mortierella gamsii VKM F-1402	15.3
Rhizopus stolonifer VKM F-399	19.0	Mortierella humilis VKM F-1494	11.3

	GLA (g/L)		ARA (g/L)
Cunninghamella echinulata VKM F-470	1.2	Mortierella alpina ATCC 32222	1.5
Cunninghamella echinulata VKM F-439	0.9	Mortierella hyalina VKM F-1854	0.7
Cunninghamella echinulata VKM F-531	0.7	Mortierella alpina UBOCC-A-112046	0.7
Absidia glauca CCM 450	0.7	Mortierella gamsii VKM F-1402	0.5
Absidia glauca CCM 451	0.7	Mortierella elongata VKM F-524	0.4
Absidia cylindrospora VKM F-1632	0.6	Mortierella gemmifera VKM F-1631	0.4
Mucor fragilis UBOCC-A-109196	0.6	Mortierella alpina UBOCC-A-112047	0.4
Mucor racemosus UBOCC-A-111127	0.6	Mortierella gemmifera VKM F-1651	0.4
Absidia cylindrospora CCM F-52T	0.5	Mortierella zonata UBOCC-A-101348	0.3
Mucor circinelloides VI 04473	0.5	Mortierella gemmifera VKM F-1252	0.3

Table S2 Total lipid content of biomass (%), total lipid concentration (g/L medium) and calculated cetane number (Ramírez-Verduzco et al.[1]) of the 10 most promising Mucoromycota strains for biodiesel production

	Total lipid (%)		Total lipid (g/L)		CN
Absidia glauca CCM 451	47.2	Cunninghamella blakesleeana CCM F-705	8.3	Umbelopsis vinacea CCM F-539	62.8
Absidia glauca CCM 450	39.1	Umbelopsis vinacea CCM F-539	7.9	Umbelopsis vinacea UBOCC-A-101347	62.3
Cunninghamella blakesleeana CCM F-705	36.7	Umbelopsis vinacea UBOCC-A-101347	7.0	Lichtheimia corymbifera CCM 8077	62.1
Absidia coerulea VKM F-627	35.6	Absidia glauca CCM 450	6.5	Rhizopus oryzae CCM 8075	61.5
Absidia glauca UBOCC-A-101330	34.8	Absidia glauca CCM 451	6.2	Cunninghamella blakesleeana CCM F-705	61.1
Umbelopsis vinacea CCM F-539	34.8	Umbelopsis isabellina VKM F-525	6.0	Umbelopsis isabellina UBOCC-A-101351	60.9
Absidia glauca CCM F-444	33.3	Absidia coerulea CCM 8230	5.3	Lichtheimia corymbifera VKM F-513	60.8
Umbelopsis vinacea CCM 8333	31.8	Cunninghamella blakesleeana VKM F-993	5.3	Umbelopsis isabellina UBOCC-A-101350	60.8
Mucor hiemalis UBOCC-A-101359	31.8	Lichtheimia corymbifera CCM 8077	4.8	Cunninghamella blakesleeana VKM F-993	60.7
Mucor hiemalis UBOCC-A-112185	31.5	Absidia glauca CCM F-444	4.6	Umbelopsis vinacea CCM 8333	60.7

Table S3 Tentative peak assignment in FTIR spectra of filamentous fungi. Abbreviations: asym, antisymmetric; sym, symmetric; str, stretching; def, deformation [2-8]

**No.**	**Frequency (cm^{-1})**	**Assignment**	**Main biomolecules**
1	~3300	N-H str (amide A), O-H str	Protein, Carbohydrate
2	3010	=C-H str	Lipid
3	2955	C-H str (asym) of $-CH_3$	Lipid
4	2925	str of $>CH_2$ of acyl chains (asym)	Lipid
5	2850	str of CH_2 of acyl chains (sym)	Lipid
6	1745	C=O str.	Lipid
7	1680-1640	Amide I band (C=O str)	Protein
8	1580-1520	Amide II (CONH bending)	Protein
9	1465	CH_2 def	Lipid
10	1410	Amide III band (C-N str)	Protein
11	1380	CH_3 bending	Lipid
12	1265	P=O str (asym) of $>PO_2$ phosphodiesters	Polyphosphate, Phospholipid
13	1155	C-O-C stretch	Lipid
14	1080	P O str (sym) of $>PO_2$	Polyphosphate, Phospholipid
15	900-1200	C-O str, C-C str., C-O-H def. C-O-C def.	Carbohydrate
16	875	P-O-P stretching	Polyphosphate, Phospholipid
17	725	CH_2 def	Lipid

Table S4 Prediction of total lipid content in fungal biomass from (preprocessed) FTIR spectra for all strains and at the genus level (*Amylomyces* and *Lichtheimia* were treated together with *Mucor* and *Absidia*, respectively)

R^2 between GC total lipid and FTIR data	Peak height [a] 1745 cm^{-1}	Peak height [b] 1745 cm^{-1}	Peak height ratio[b] 1745/1655 cm^{-1}	PLSR [c] (factors/RMSECV)
**All (100)**	0.41	0.47	0.22	0.72 (3/4.20)
***Mucor*/*Amylomyces* (43)**	0.36	0.42	0.46	0.80 (6/2.72)
***Rhizopus* (9)**	0.31	0.54	0.50	0.73 (2/1.80)
***Umbelopsis* (9)**	0.04	0.07	0.00	0.43 (8/2.60)
***Absidia*/*Lichtheimia* (13)**	0.44	0.21	0.12	0.62 (9/3.75)
***Cunninghamella* (5)**	0.14	0.17	0.01	0.77 (5/2.27)
***Mortierella* (21)**	0.37	0.66	0.15	0.78 (10/3.45)

a EMSC correction with linear and quadratic component were used in the full spectral range (4000-500 cm^{-1})

b Second derivative spectra were obtained by the Savitzky–Golay (S-G) algorithm using windows size (ws) 15 and a second degree polynomial in the full spectral range (4000-500 cm^{-1}) followed by EMSC

c Spectra were prepocessed with (S-G) algorithm (ws 15, 2nd degree polynomial) followed by EMSC and cross-validation with leave-one-biological-replicate-out method. The maximum amount of PLSR components was 10. RMSECV: Root Mean Square Error of Cross Validation

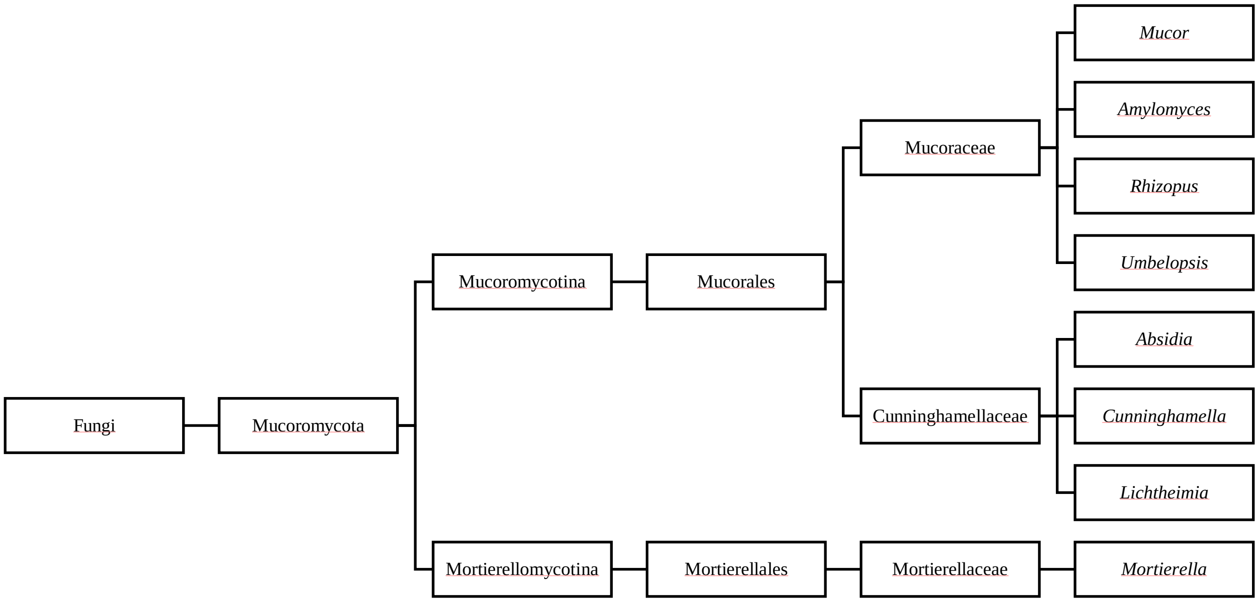

Fig. S1 Classification of Mucoromycota fungi used in the study (according to Westerdijk Fungal Biodiversity Institute [9])

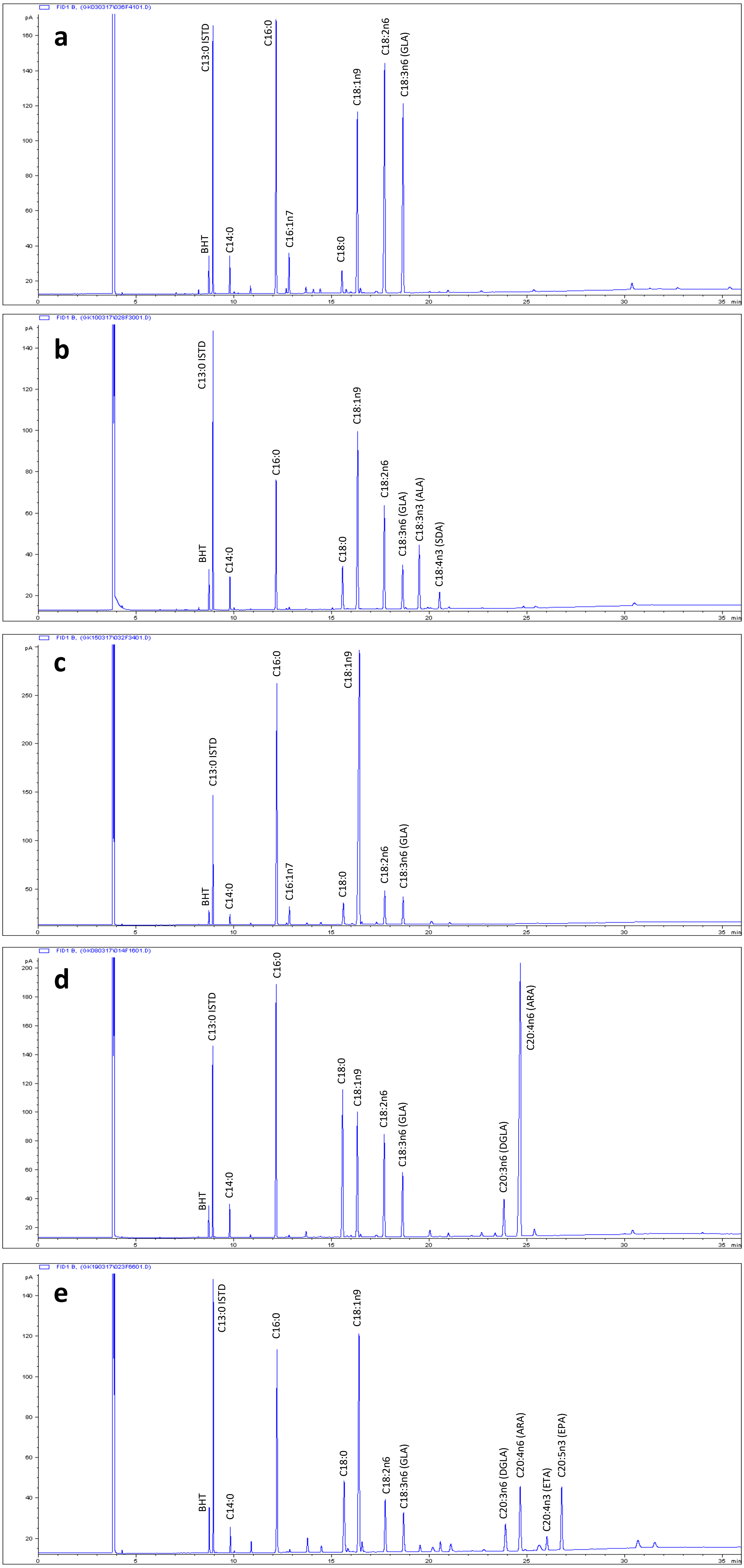

Fig. S2 GC-FID chromatograms of Mucoromycota fungi fatty acid composition (examples) **a)** *Mucor fragilis* UBOCC-A-109196, **b)** *Mucor flavus* CCM 8086 (15°C) **c)** *Umbelopsis vinacea* CCM F-539, **d**) *Mortierella alpina* ATCC 32222, **e)** *Mortierella humilis* VKM-1494 (15°C). BHT: butylated hydroxytoluene (antioxidant)

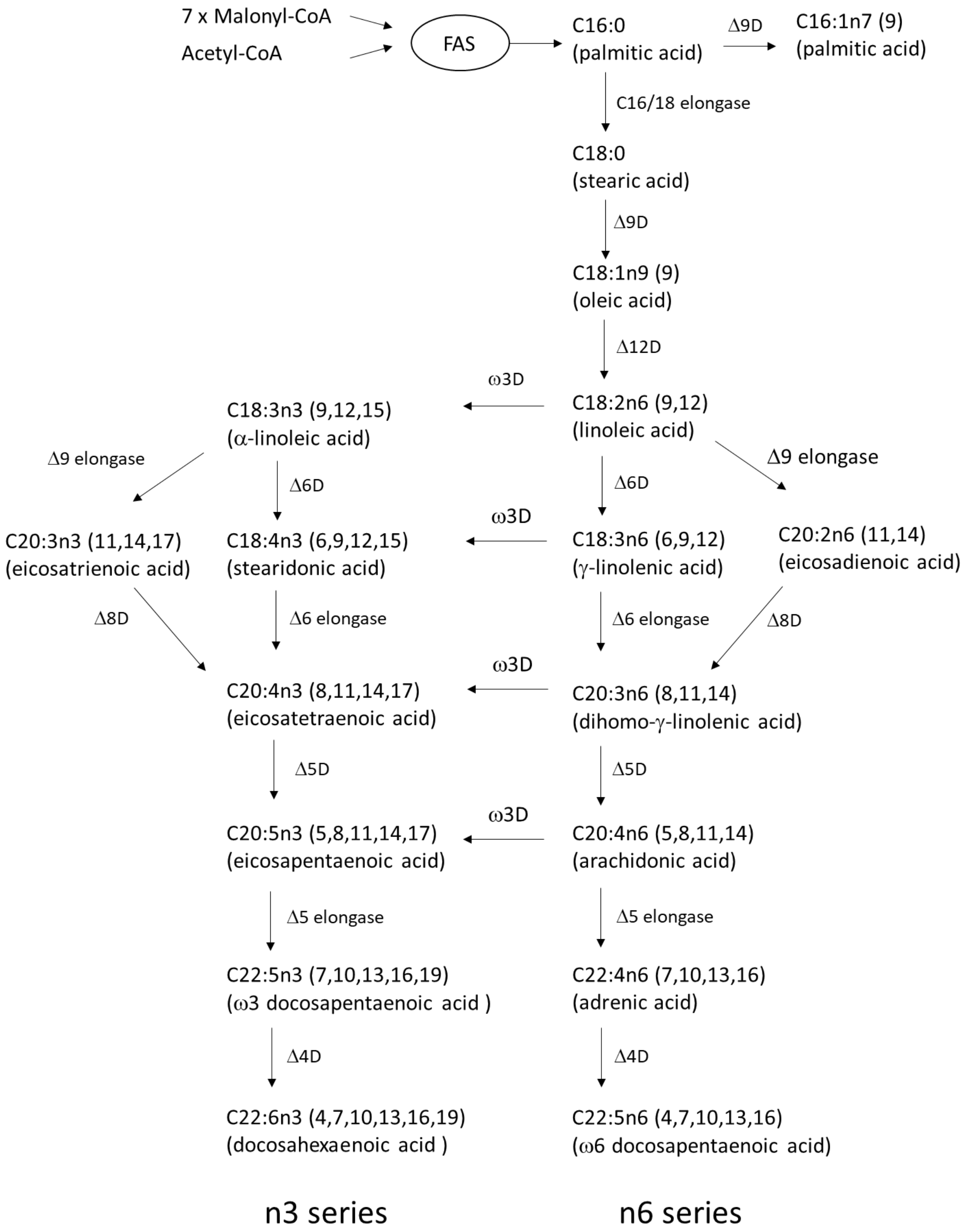

Fig. S3 Pathways for the formation of PUFA in microorganisms. FAS: fatty acid synthase, D: desaturase, Δ: position of double bond from carboxylic end. Adapted from Ratledge, 2004 [10], Lee *et al.*, 2016 [11], and Ruiz-López *et al.*, 2012 [12]

References:

1. Ramírez-Verduzco LF, Rodríguez-Rodríguez JE, del Rayo Jaramillo-Jacob A: **Predicting cetane number, kinematic viscosity, density and higher heating value of biodiesel from its fatty acid methyl ester composition.** *Fuel* 2012, **91:**102-111.
2. Ami D, Posteri R, Mereghetti P, Porro D, Doglia SM, Branduardi P: **Fourier transform infrared spectroscopy as a method to study lipid accumulation in oleaginous yeasts.** *Biotechnology for biofuels* 2014, **7:**12.
3. Beekes M, Lasch P, Naumann D: **Analytical applications of Fourier transform-infrared (FT-IR) spectroscopy in microbiology and prion research.** *Veterinary microbiology* 2007, **123:**305-319.
4. Carosio F, Alongi J, Malucelli G: **Layer by layer ammonium polyphosphate-based coatings for flame retardancy of polyester–cotton blends.** *Carbohydrate Polymers* 2012, **88:**1460-1469.
5. Guillén MD, Cabo N: **Relationships between the composition of edible oils and lard and the ratio of the absorbance of specific bands of their Fourier transform infrared spectra. Role of some bands of the fingerprint region.** *Journal of Agricultural and Food Chemistry* 1998, **46:**1788-1793.
6. Lü F, Shao L-M, Zhang H, Fu W-D, Feng S-J, Zhan L-T, Chen Y-M, He P-J: **Application of Advanced Techniques for the Assessment of Bio-stability of Biowaste-derived Residues: A Minireview.** *Bioresource Technology* 2017.
7. Miglio R, Palmery S, Salvalaggio M, Carnelli L, Capuano F, Borrelli R: **Microalgae triacylglycerols content by FT-IR spectroscopy.** *Journal of applied phycology* 2013, **25:**1621-1631.
8. Szeghalmi A, Kaminskyj S, Gough KM: **A synchrotron FTIR microspectroscopy investigation of fungal hyphae grown under optimal and stressed conditions.** *Analytical and bioanalytical chemistry* 2007, **387:**1779-1789.
9. [http://www.mycobank.org/]
10. Ratledge C: **Fatty acid biosynthesis in microorganisms being used for single cell oil production.** *Biochimie* 2004, **86:**807-815.
11. Lee JM, Lee H, Kang S, Park WJ: **Fatty acid desaturases, polyunsaturated fatty acid regulation, and biotechnological advances.** *Nutrients* 2016, **8:**23.
12. Ruiz-López N, Sayanova O, Napier JA, Haslam RP: **Metabolic engineering of the omega-3 long chain polyunsaturated fatty acid biosynthetic pathway into transgenic plants.** *Journal of experimental botany* 2012, **63:**2397-2410.
